# Haematological reference intervals of sows at end gestation in ten French herds, the impact of parity on haematological parameters and the consequences on reproductive performance

**DOI:** 10.1186/s40813-021-00227-w

**Published:** 2021-08-17

**Authors:** Gwenaël Boulbria, Charlotte Teixeira Costa, Valérie Normand, Véronique Bachy, Daphné Rochel, Mathieu Brissonnier, Pauline Berton, Franck Bouchet, Arnaud Lebret

**Affiliations:** 1 Porc.Spective Swine Vet Practice, ZA de Gohélève, rue Joseph et Étienne Montgolfier, 56920 Noyal- Pontivy, France; 2 Rezoolution Pig Consulting Services, ZA de Gohélève, rue Joseph et Étienne Montgolfier, 56920 Noyal- Pontivy, France; 3 Orbio - Finalab Veterinary Laboratories Group, 12, rue du 35 ème régiment d’aviation, 69500 Bron, France

**Keywords:** Pig, Blood, Haematology, Reference interval, Haemoglobin

## Abstract

**Background:**

Changes in haematological values occur during the reproductive cycle. In veterinary swine practice, haematological reference intervals for this period are scarce. Over past decades, there has been a remarkable increase in reproductive prolificacy, possibly making previously established haematological reference intervals for sows outdated.

**Objectives:**

The aim of this study was to provide updated haematological reference intervals for sows at end-gestation, to study the influence of parity on those haematological parameters and to evaluate the impact of haemoglobin levels on production performance.

**Methods:**

The data presented in this article were obtained using blood samples from 198 apparently healthy and conventionally managed group-housed sows at end-gestation from ten breeding herds located in France. The samples were analysed for haematological variables using impedance technique on Horiba ABX analyser (Horiba, Kyoto, Japan). The reference intervals were calculated according to the guidelines of The American Society for Veterinary Clinical Pathology using SUMMARY procedure in R Studio. Analysis of variance (ANOVA) models were used to evaluate the influence of parity on each haematological parameter and the impact of haemoglobin values on production performances at farrowing. Differences were considered as significant if *p* < 0.05.

**Results:**

Reference intervals produced in this study were similar to previously published references but we noticed marked differences in white blood cell values. The study of the impact of parity revealed significant changes for gilts and parity 5 + sows regarding haematological values. Gilts had higher red and white blood cells counts, haemoglobin values and haematocrit values. Regarding haemoglobin values, the higher the number of liveborn and weaned piglets per litter, the lower the haemoglobin value at end-gestation. For sows of fifth or higher gestation, we found that the higher the percentage of stillborn piglets, the lower the haemoglobin value at end-gestation.

**Conclusions:**

This study provides haematological reference intervals for sows at end-gestation. These will be useful for swine veterinarians and researchers for a better understanding of the influence of parity on haematological parameters and haemoglobin values and their relation to reproductive performance.

## Background

Despite their wide availability, haematological values are rarely used in practice for the evaluation of sow health and its potential consequences on reproductive performance. Up to now, very few haematological variables have been used in swine practice because of the limited availability of reference intervals [1]. Moreover, over past decades, there has been a marked increase in litter size and reference intervals that were previously established may now be outdated. Haematological parameters play a significant role on swine reproductive performance. In particular, the impact of low haemoglobin concentration on the risk of stillborn piglets [2] has already been demonstrated. Haematological values of swine are influenced by pathological processes and by a wide range of environmental and physiological factors including diet, age, sex, housing and management practices [3]. Many infectious agents can cause changes in white blood cell (WBC) counts, as well as red blood cell (RBC) counts, haemoglobin concentration (Hb) and haematocrit (Hct), depending on the severity of clinical signs and magnitude of the immune response [4]. Commonly cited agents inducing changes in haematological parameters in pigs are infection with *Mycoplasma suis* causing anaemia [5] and the porcine reproductive and respiratory syndrome (PRRS) virus causing a depletion of WBC [6].

The measurement of haematological parameters in sows can serve as a practical tool for assessing pathological conditions and reflect metabolic disturbances caused by inappropriate feeding or nutritional deficiencies. Several studies have investigated the influence of age and stage of gestation on the variability of blood values [4, 7, 8]. Indeed, physiological differences in haematological parameters have been observed depending on the parity of the sow and litter size, with an increased litter size associated with anaemia [9]. The aim of this study was to produce haematological reference intervals for sows at end-gestation. The second objective was to evaluate the influence of parity on haematological values and to provide an overview of the impact of haemoglobin concentration on reproductive performance.

## Materials and methods

### Farms and animals

Blood samples were collected five to twelve days before farrowing from 199 sows belonging to two farrow-to-wean and eight farrow-to-finish farms in Brittany (France). Selected herds were epidemiologically unrelated (different nucleus herds for gilts and different food suppliers). The breed of the sows was either Large White x Landrace (eight farms) or Large White x Landrace x Tai Zumu (two farms). Farms were also selected for the absence of infectious outbreaks in sows or suckling piglets and the absence of collective antibiotic treatments of sows in the last six months prior to the study. At sampling time, sows were group-housed in indoor facilities in accordance with the European Union legislation and were free from clinical disease based on veterinary observation. Herds were enrolled and all samples and data were collected between December 2017 and March 2018. Farm status regarding PRRSV and PCV-2 is presented in Table 1. PRRSV status has been determined according to the AASV classification [10]. All positive stable herds were vaccinated with modified live vaccines. Finally, for each farm, parasitic status was unknown. On each farm, 20 sows from the same batch were sampled 12 to 5 days before farrowing. In one herd, only 19 sows could be sampled. The samples were stratified on the basis of gestation rank: 4 to 6 pregnant gilts, 3 to 6 sows in second gestation, 3 to 6 sows in third or fourth gestation and 4 to 9 sows in fifth or higher gestation.

**Table 1:** Farm status regarding main diseases affecting haematological parameters

Farm	PRRSV status [10]	PRRSV vaccination	PCV-2 gilts vaccination	PCV-2 multiparous vaccination
1	Negative	-	Yes	No
2	Positive stable	Yes	No	No
3	Positive stable	Yes	Yes	No
4	Negative	-	No	No
5	Positive stable	Yes	Yes	Yes
6	Negative	-	No	No
7	Negative	-	No	No
8	Positive stable	Yes	No	No
9	Negative	-	Yes	Yes
10	Positive stable	Yes	No	No

### Blood sample collection and analyses

Sows were restrained using a wire nose snare. Blood samples (5 ml) were obtained by venipuncture of the jugular vein and collected into EDTA tubes. Blood samples were stored under positive cold conditions until submission to the diagnostic laboratory Orbio (Finalab, Bron, France) within 24 h. Standard haematological evaluations were performed using the impedance technique on a Horiba ABX analyser (Horiba, Kyoto, Japan). The following haematological variables were measured: red blood cells (RBC) count, haematocrit (Hct), haemoglobin concentration (Hb), mean corpuscular volume (MCV), mean corpuscular haemoglobin (MCH), mean corpuscular haemoglobin concentration (MCHC), platelet count (PLT), white blood cells (WBC) count, and differentiation of neutrophils (absolute count and percentage), eosinophils (absolute count and percentage), monocytes (absolute count and percentage) and lymphocytes (absolute count and percentage).

### Performance data collection

Numbers of total born, number of stillborn piglets and number of piglets weaned per sow were recorded by the farmer. Piglets were considered stillborn by visual judgement of the farmer and corresponded to dead piglets collected during or immediately after farrowing. No necropsy of stillborn piglets was performed.

### Statistical analysis

The reference intervals were calculated according to the guidelines of the American Society for Veterinary Clinical Pathology [11] (ASVCP) using the SUMMARY procedure in RStudio. Each of the variables was checked for the presence of extreme outliers and those outliers were removed from the analysis. Next, the data were submitted to reference limit evaluations. The 95 %-percentile reference intervals method (2.5th and 97.5th percentile) was used to determine the reference intervals as recommended by ASVCP for a sample size over 120. The reference intervals with 90 %-confidence limits were calculated with Reference Value Advisor (V.2.1) [12] freeware in Excel (Microsoft Corporation, Redmond, USA). Then, ANOVA models were used to evaluate the influence of farm and parity for each haematological parameter and the impact of haemoglobin values on production performances at farrowing. The statistical analyses were carried out using RStudio version 4.0.2 (R Core Team, 2020). Differences were considered statistically significant if *p* < 0.05.

## Results

A total of 199 sows were sampled, including 49 pregnant gilts, 47 s parity sows, 52 third or fourth parity sows and 51 sows with parity five or higher. One sow was excluded from the study because haematological measures were not possible due to sample haemolysis, so that a total of 198 sows were included in the analysis in order to produce the reference intervals presented hereafter. For each criterion, the number of outlier values removed to produce reference intervals is presented in Table 2.

**Table 2:** Number of values included for reference intervals after the removal of outliers

Variables	Number of values	Number of outliers	Number of values included for reference intervals
RBC	198	4	194
Hb	198	6	192
HCT	198	1	197
MCV	198	0	198
MCH	198	3	195
MCHC	198	3	195
WBC	198	1	197
Neutrophils	198	1	197
Eosinophils	198	2	196
Monocytes	198	1	197
Lymphocytes	198	2	196
PLT	198	0	198

N indicates number of animals, *RI *reference interval, *RBC *red blood cell count, *WBC *white blood cell count, *Hb *haemoglobin, *HCT *haematocrit, *MCV *mean corpuscular volume, *MCH *mean corpuscular haemoglobin, *MCHC *mean corpuscular haemoglobin concentration, *PLT *platelet count

### Reference intervals for 198 end-gestation sows

Based on a non-parametric test regarding individual blood samples, the time between sampling and farrowing, the farm were set as a random effect. The reference intervals (RI) for RBC, WBC and platelets are listed in Table 3. We compared mean haematological values from our study with previously published RIs in Table 4. We noticed some marked differences (> 10 %), with lower WBC counts and the percentage of lymphocytes, and a higher percentage of neutrophils in our study.

**Table 3:** Reference intervals for 198 clinically healthy sows at end-gestation in ten French herds

Variables	Unit	N	Mean	Median	SD	Range (min-max)	RI with 90% Confidence Interval (CI)	
							Lower limit of CI^**a**^	Upper limit of CI^**a**^
RBC	10^12^/L	198	5.8	5.8	0.7	3.49-7.75	4.3 (4.0-4.7)	7.2 (6.9-7.4)
Hb	g/dL	198	12.1	12.1	1.2	7.7-15.4	9.4 (8.6-10.2)	14.5 (8.6-10.2)
HCT	%	198	39.7	39.6	4.6	11.6-54.8	30.9 (24.7-32.5)	48.6 (46.4-50.1)
MCV	fl	198	68.6	68.0	3.3	60-77	61.0 (60.0-63.0)	75.0 (74.0-76.0)
MCH	pg	198	21.0	20.9	1.2	17.4-24.7	18.2 (17.8-19.0)	23.2 (23.1-24.4)
MCHC	g/dL	198	30.5	30.6	0.9	24.5-33.6	29.1 (26.2-29.4)	31.8 (31.6-33.5)
WBC	10^9^/L	198	12.1	11.9	2.7	3.1-22.3	7.6 (4.1-8.3)	18.8 (17.1-19.3)
Neutrophils	10^9^/L	198	7.2	7.0	1.8	0.3-14.4	3.5 (0.4-4.8)	11.9 (10.7-12.9)
	%	198	59.3	60.4	8.2	7.7-76.7	47.7 (11.6-49.6)	72.0 (69.7-74.0)
Eosinophils	10^9^/L	198	1.0	0.9	0.5	0-2.6	0.3 (0-0.3)	2.2 (1.9-2.4)
	%	198	7.7	7.1	3.4	0.4-17	2.9 (0.5-3.3)	15.7 (13.9-16.4)
Monocytes	10^9^/L	198	0.6	0.6	0.2	0.1-1.5	0.3 (0.2-0.4)	0.9 (0.9-1.1)
	%	198	4.8	4.8	0.9	1.9-8.3	3.3 (2.8-3.6)	6.8 (6.2-7.3)
Lymphocytes	10^9^/L	198	3.3	3.3	0.9	1.3-6.7	1.9 (1.7-1.9)	5.6 (5.1-6.1)
	%	198	28.2	27.4	8.3	10.3-83.5	17.3 (16.1-19.1)	40.5 (37.5-82.0)
PLT	10^9^/L	198	225.8	221.5	74.5	42-441	90.7 (72-109)	400.7 (346-434)

N indicates number of animals, *RI* reference interval, *RBC* red blood cell count, *WBC* white blood cell count, *Hb* haemoglobin, *HCT* haematocrit, *MCV* mean corpuscular volume, *MCH* mean corpuscular haemoglobin, *MCHC* mean corpuscular haemoglobin concentration, *PLT* platelet count

^a^ using 2.5th to 97.5th inter-percentile range

**Table 4:** Mean haematological variables of our study compared with the literature

Variables	Unit	Mean	Thorn CE., 2010 [2]	Ježek et al., 2018 [3]	Bhattarai et al., 2019 [4]
Stage		5-12 days before parturition	2 weeks or less before parturition	Gestation	Mid-gestation (between 56-70 days of gestation)
RBC	10^12^/L	**5.8**	5.7	5.62	6.14
Hb	g/dL	**12.1**	12.6	11.6	12.03
HCT	%	**39.7**	40.0	34.9	-
MCV	fl	**68.6**	70.0	62.2	63.4
MCH	pg	**21.0**	-	20.6	-
MCHC	g/dL	**30.5**	31.6	33.2	31.04
WBC	10^9^/L	**12.1**	15.6	15.21	15.44
Neutrophils	%	**59.3**	39.0	-	38.4
Eosinophils	%	**7.7**	0.7	7.0	7.4
Monocytes	%	**4.8**	6.0	1.0	4.2
Lymphocytes	%	**28.2**	52.0	43.0	47.8
PLT	10^9^/L	**225.8**	-	274	-

*RBC* red blood cell count, *Hb* haemoglobin, *HCT* haematocrit, *MCV* mean corpuscular volume, *MCH* mean corpuscular haemoglobin, *MCHC* mean corpuscular haemoglobin concentration, *WBC* white blood cell count, *PLT* platelet count

### Influence of parity rank on haematological parameters

Mean, minimum and maximum reference values per parity rank are presented in Table 5. Mean RBC, Hb, Hct, WBC and PLT values were significantly higher in gilts, while mean MCV and MCH values were significantly lower. Mean haematological values of sows in parity 5 or higher were also significantly different from other parity ranks for RBC, MCV, MCH, MCHC, WBC and PLT.

**Table 5:** Haematological values per parity ranks presented as mean (minimum-maximum)

Variables	Units	Gilts ***N*** = 49	Sows		
			Parity 2 ***N*** = 47	Parity 3-4 ***N*** = 52	Parity 5 or more ***N*** = 51
RBC	10^12^/L	6.2 (3.5-7.4)^a^	5.9 (4.3-7.8)^b^	5.7 (4.6-7.2)^b^	5.5 (4.1-6.4)^b^
Hb	g/dL	12.5 (7.7-15.4)^a^	12.2 (8.7-14.9)^a^	11.9 (8.6-14.0)^a^	12.0 (9.4-14.5)^a^
HCT	%	41.2 (24.7-54.8)^a^	39.8 (30.9-50.1)^a^	38.8 (11.6-45.8)^a^	38.8 (30.2-48.3)^a^
MCV	fl	62.32 (60-73)^a^	67.97 (61-74)^ab^	68.71 (60-76)^b^	71.06 (66-77)^c^
MCH	pg	20.28 (17.8-23.2)^a^	20.86 (18.4-24.4)^a^	20.85 (17.4-22.5)^a^	21.96 (20.1-24.7)^b^
MCHC	g/dL	30.53 (29.1-31.7)^ab^	30.68 (27.1-33.6)^ab^	30.29 (30-31.8)^a^	30.93 (29.2-33.5)^b^
WBC	10^9^/L	13.9 (8.3-22.3)^a^	12.1 (8.2-18.9)^b^	11.5 (3.1-16.7)^bc^	10.9 (4.1-18.8)^c^
PLT	10^9^/L	250.4 (115-428)^a^	240.9 (100-323)^ab^	216.3 (72-434)^ab^	197.4 (42-441)^b^

*RBC* red blood cell count, *Hb* haemoglobin, *HCT* haematocrit, *MCV* mean corpuscular volume, *MCH* mean corpuscular haemoglobin, *MCHC* mean corpuscular haemoglobin concentration, *WBC* white blood cell count, *PLT* platelet count

^a,b,c^ Different letters in superscript within a line means *p* < 0.05

### Impact of haemoglobin concentration on farrowing performance parameters

**Fig. 1:**
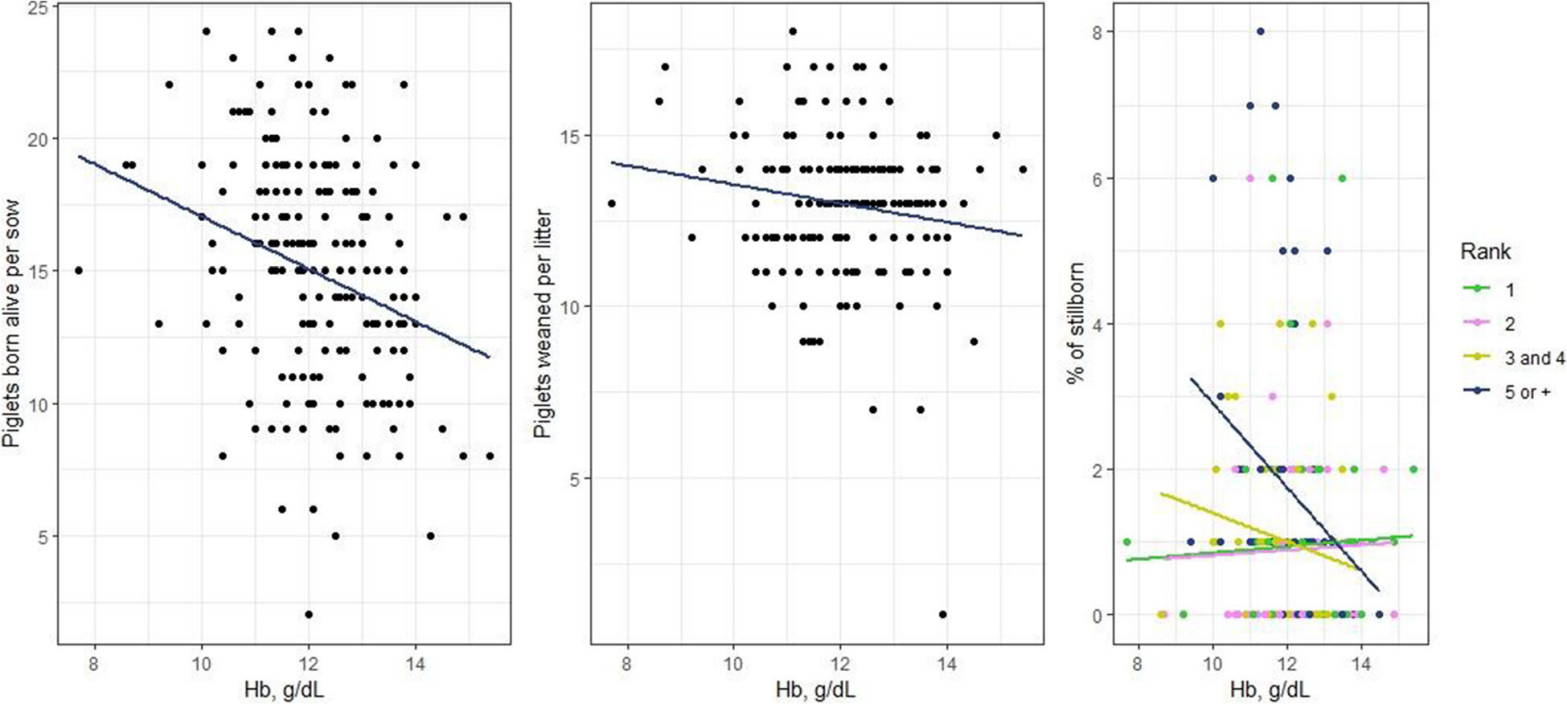
Correlations between haemoglobin concentration and performance parameters. The blue lines for piglets born alive and pigs weaned per sow indicate there were significant differences between haemoglobin concentration and number of piglets born alive or weaned per sow regardless of parity rank (*p* < 0.05). The blue line for stillbirth in sows or fifth gestation or more indicate also a significant difference (*p* < 0.05)

In the studied population, sows had an average of 14.9 liveborn piglets and 12.9 weaned piglets per litter. Concerning associations between haemoglobin concentration and farrowing performance, a significant inverse relationship was observed; the higher the number of liveborn piglets, the lower the haemoglobin value at end-gestation, regardless of parity rank (Fig. 1). We observed the same relationship between haemoglobin concentration and number of weaned piglets per litter.

In the studied population, an average of 6.7 % of piglets were stillborn. This percentage was relatively low compared to the average in France (7.4 % in 2018). For sows in their fifth or higher gestation, the lower the haemoglobin value at end-gestation, the higher the percentage of stillborn piglets (*p* = 0.002) (Fig. 1). For the other parity ranks, the difference was not significant.

## Discussion

Assuming that haematological data undergo constant change due to genetic progress and an increase in litter size, the aims of this study were to propose updated RI for French sows at end-gestation, to study the impact of parity ranks on haematological parameters and to determine whether haemoglobin concentration has an influence on performance parameters at farrowing.

We sampled 199 sows, a population size consistent with previous studies. In this study, samples were obtained by venipuncture at the jugular vein, the most common sampling site in practice. Porcine RBC are relatively fragile and turbulence or improper handling of the sample frequently results in haemolysis [13]. In our study, blood samples were immediately stored under positive cold conditions and sent to the laboratory within 24 h. Using this protocol, only one sample was excluded because of haemolysis. We determined RIs according to the ASVCP guidelines, and the final sample size was amply sufficient to establish these [11, 14].

Haematological reference intervals can vary according to breed [15], season [13], physiological status [4], age [4], management factors [4] and laboratory measurement techniques. We randomly selected ten different herds that were epidemiologically unrelated and had different management practices, so that the population studied could be considered as a representative sampling of French sows. Farm was a significant source of variation for most haematological parameters in a previous study [16], but this was not the case in our study. Similarly, the time lag between sampling and farrowing (5–12 days in our study) was not a variable factor for RIs. All sows were sampled during winter to avoid a seasonal effect. We sampled sows according to parity ranks in order to approximate the demography as described of French herds.

The differences observed between our results and previously published RIs could be explained by several hypotheses. The maximum time between sampling and haematological evaluation was 24 h in our study and samples were stored immediately under positive cold conditions after sampling until delivery to the laboratory. Routine haemogram values are reported to be stable at 4 °C for up to 36 h, but the WBC differentials tend to become less reliable after 12 h of collection [17]. Furthermore, the methodology used to produce RIs could differ from the one used in this study.

Unfortunately, although there is a wide range of RIs for swine today, those involving sows during the same physiological stage are rare; it is therefore difficult to compare our data with those previously described in the literature, mainly because of the gestation stage of sows at sampling time. First, it was described that approximately two weeks before parturition, RBC numbers in sows decrease and continue to do so until the end of lactation [13]. Secondly, the total WBC number decreases during gestation [13]. For these reasons, comparisons of RIs between studies must be undertaken with care, and take into account the sampling and laboratory protocols and the gestation stage at sampling.

Keeping these limitations in mind, we compared our haematological data with three earlier studies performed by Catherine Thorn [18] (who investigated sows two weeks or less before parturition), by Jožica Ježek [4] (who presented haematological values for 224 pregnant sows) and Sheeva Bhattarai [1] (who studied 248 sows at mid-gestation using the ASVCP guidelines to produce RIs). Even if variations could be observed in both haematologic cell lines, red blood cell composites were found to differ little during gestation compared to white blood cell composites [18]. WBC and leukocyte parameters were previously reported to be closely related to the health status of the sow and the conditions of sampling and samples processing [13]. The increase in WBC may be explained by the high level of neutrophils in our population. Apart from the hypothesis of the poor reliability of WBC count because of the time lag between sampling and analysis, we suspect that stress at the time of sampling might have affected our results. It has been already described in other species like cats and dogs [18]. Stress is one of the factors that can cause neutrophilia and lymphopenia, as observed in our study. Indeed, use of a wire nose snare can cause stress during blood sampling. We sampled three tubes per animal, so each sow was restrained for three to five minutes. Subclinical disease could also have caused an altered WBC count. For example, viral diseases like Porcine Reproductive and Respiratory Syndrome virus [19] and porcine circovirus type 2 (PCV-2) [20] have been associated with lymphopenia in previously published studies. Moreover, neutrophilia has previously been described in pigs infected with PCV-2 [21]. In this study, all sampled sows were clinically healthy and came from PRRSV negative or positive stable herds. Subclinical infection with PCV-2 was not assessed during our study which could be a limitation. Finally, the percentage of eosinophils was consistent with that found by Sheeva Bhattarai and Jožica Ježek but we observed a marked difference with Catherine Thorn. In our study, all sows are group-housed during gestation in accordance with European Union law. Group housing is a risk factor for parasitic infestation (e.g. roundworm), possibly resulting in higher eosinophil counts than those found in older studies.

In this study, we compared the impact of parity ranks on haematological values and we found significant differences for all criteria tested, except for MCHC, between gilts and higher parities. The impact of parity has already been demonstrated for Hb values in previous studies: Hb values decreased after first farrowing [2, 9]. Results presented in the more recent study are consistent with our results for Hct too: Hct values decreased after first parity. Our results highlight the fact that age and parity of sows in particular has an impact on haematological values. Gilts should be considered independently when interpreting haematological results.

In the last part of our study, we focused on the impact of haemoglobin concentration on the reproductive performance of sows. Considering only sow’s haemoglobin levels could be a limitation. In a Canadian study, no association was observed between stillbirth and the sow’s haemoglobin, although an association between the probability of stillbirth and reduced Hb in piglets was found [22]. In our study, we observed a significant relation between the sow’s Hb and the percentage of stillborn piglets for sows after their fifth or higher gestation. High haemoglobin concentration in the sow’s blood may explain the efficiency of uterine contractions and the vigour of the litter at parturition [2]. This might have a positive effect on the number of liveborn piglets. Alternatively, a previous study suggested that a possible explanation for the association between Hb and stillbirth could be the decrease in oxygen supply to the piglets due to low iron status in the sow [23]. We can compare our results with two previously published studies. The first one showed no relationship between the number of stillborn piglets and haemoglobin values, measured using the rapid HemoCue® Hb 201 + tool [9]. The second study sampled sows at the time of farrowing. They found a significant association between stillbirth and Hb, regardless of the parity of the sows. In the study, all dead piglets collected during farrowing were necropsied to determine whether they were stillborn or not using the lung flotation technique [2]. This was not performed in our study, where stillbirth was based only on the subjective judgement of the farmer.

Other performance parameters at farrowing were analysed in our study. The higher the number of liveborn and weaned piglets, the lower the haemoglobin concentration. Logically, we assumed that the higher is the number of liveborn, the higher the number of weaned piglets, which could explain the relationship between haematological status at late gestation and the number of weaned piglets. A significant link between body reserves and haemoglobin concentration has been demonstrated previously. Regardless of parity rank, haemoglobin concentration was lower in sows with a lower backfat thickness [9]. Unfortunately, in our study, we did not take into consideration body reserves.

## Data Availability

All datasets used in this study are available from the corresponding author on reasonable request.
